# Laccase Immobilized onto Zirconia–Silica Hybrid Doped with Cu^2+^ as an Effective Biocatalytic System for Decolorization of Dyes

**DOI:** 10.3390/ma12081252

**Published:** 2019-04-16

**Authors:** Katarzyna Jankowska, Filip Ciesielczyk, Karolina Bachosz, Jakub Zdarta, Ewa Kaczorek, Teofil Jesionowski

**Affiliations:** Institute of Chemical Technology and Engineering, Faculty of Chemical Technology, Poznan University of Technology, Berdychowo 4, PL-60965 Poznan, Poland; katarzyna.a.antecka@doctorate.put.poznan.pl (K.J.); filip.ciesielczyk@put.poznan.pl (F.C.); karolina.h.bachosz@doctorate.put.poznan.pl (K.B.); jakub.zdarta@put.poznan.pl (J.Z.); ewa.kaczorek@put.poznan.pl (E.K.)

**Keywords:** inorganic oxide materials, surface functionalization, enzyme immobilization, laccase, dyes decolorization

## Abstract

Nowadays, novel and advanced methods are being sought to efficiently remove dyes from wastewaters. These compounds, which mainly originate from the textile industry, may adversely affect the aquatic environment as well as living organisms. Thus, in presented study, the synthesized ZrO_2_–SiO_2_ and Cu^2+^-doped ZrO_2_–SiO_2_ oxide materials were used for the first time as supports for laccase immobilization, which was carried out for 1 h, at pH 5 and 25 °C. The materials were thoroughly characterized before and after laccase immobilization with respect to electrokinetic stability, parameters of the porous structure, morphology and type of surface functional groups. Additionally, the immobilization yields were defined, which reached 86% and 94% for ZrO_2_–SiO_2_–laccase and ZrO_2_–SiO_2_/Cu^2+^–laccase, respectively. Furthermore, the obtained biocatalytic systems were used for enzymatic decolorization of the Remazol Brilliant Blue R (RBBR) dye from model aqueous solutions, under various reaction conditions (time, temperature, pH). The best conditions of the decolorization process (24 h, 30 °C and pH = 4) allowed to achieve the highest decolorization efficiencies of 98% and 90% for ZrO_2_–SiO_2_–laccase and ZrO_2_–SiO_2_/Cu^2+^–laccase, respectively. Finally, it was established that the mortality of *Artemia salina* in solutions after enzymatic decolorization was lower by approx. 20% and 30% for ZrO_2_–SiO_2_–laccase and ZrO_2_–SiO_2_/Cu^2+^–laccase, respectively, as compared to the solution before enzymatic treatment, which indicated lower toxicity of the solution. Thus, it should be clearly stated that doping of the oxide support with copper ions positively affects enzyme stability, activity and, in consequence, the removal efficiency of the RBBR dye.

## 1. Introduction

Laccases are oxidoreductases which catalyse the oxidation of a wide variety of organic compounds, including mono-, di- and polyphenols as well as aliphatic and aromatic amines [1]. These enzymes are widespread in nature and most commonly extracted from white or red rot fungi, such as *Trametes versicolor* or *Trametes vilosa* [2]. Four adjacent copper atoms are located at the active sites of laccase which correspond to the blue color to the enzyme molecule, hence the protein is often called “blue oxidase”. The mechanism of reactions catalysed by laccases involves the oxidation of the substrate molecule to radicals and simultaneous reduction of the oxygen molecule into two water molecules [3]. Due to wide substrate specificity, laccases are used in many industrial processes, such as wood pulp delignification, purification of contaminated water and wastewater treatment as well as in bioremediation and decolorization of textile dye effluents [4].

The high amount of pollutants results in increasing interest in searching for new methods of their removal. Methods such as adsorption, sedimentation, coagulation, membrane techniques or photocatalysis are used for this purpose. However, these techniques are characterized by insufficient efficiency [5,6]. Therefore, the microbiological and enzymatic degradation of harmful compounds is increasingly employed. Unfortunately, due to the properties of native enzymes, such as low stability, narrow pH and temperature range of high catalytic activity, there is a need to use methods for their improvement [7]. It should be emphasized that various strategies are used to improve the thermal and chemical stability of the biocatalysts as well as to prolong their activity and facilitate their reusability, such as protein engineering, chemical modification or enzyme immobilization (which is used most frequently) [8,9]. Immobilization enhances the feasibility of the process, its economy as well as purity of the products [10,11]. Moreover, it is possible to carry out one-step immobilization and simultaneous purification of enzymes [12]. Immobilized metal ion affinity chromatography (IMAC) is also an interesting method of enzyme purification, which is based on covalent binding of the chelating compounds onto chromatographic support. The entrapment of metal ions and their high affinity to enzyme facilitate rapid peptide purification and production of heterogeneous biocatalysts. Due to its simplicity and relatively low costs, this method is used with increasing frequency in various branches of the industry [13,14]. Nevertheless, it should be clearly stated that only a proper realization of immobilization process increases enzyme rigidity or generates a stabilizing and protective microenvironment for the biomolecules. In this regard, multipoint enzyme immobilization which improves the biocatalytic properties of biomolecules by providing stable enzyme–matrix interactions and reducing subunit dissociation is crucial, particularly for multimeric enzymes such as laccases and most of the dehydrogenases [15]. Additionally, other enzyme features such as selectivity, specificity, purity, resistance to inhibitors and stability at harsh environmental conditions may also be improved [16,17,18]. Furthermore, the immobilization of biocatalysts allows to practically use the enzymes for degradation of pollutants, which has recently gained great scientific interest [19]. For instance, Koloti et al. [20] immobilized laccase onto hyperbranched polyethyleneimine/polyethersulfone (HPEI/PES) electrospun nanofibrous membrane and used this system for removal of bisphenol A. An approach for the removal of the same compound, which included immobilization of laccase onto *Hippospongia communis* spongin scaffolds, was proposed by Zdarta et al. [21]. Nevertheless, it should be noted that not only phenol and its derivatives may be degraded using immobilized laccases. Interesting examples have been presented by Bayramoglu et al. and Gioia et al., which were focused on the immobilization of laccase onto poly(hydroxyethyl methacrylate-co-vinylene carbonate) and thiolsulfinate-agarose, respectively. The obtained systems were used for degradation of Cibacron Blue 3GA, Acid Red 88 and Acid Black 172 [22,23]. It should also be underlined that proper selection of a suitable support has a significant impact on the effectiveness of the immobilization as well as on the final properties of the produced biocatalytic system. Moreover, the industrial processes carried out at various conditions, e.g., at high temperature and pressure or in the presence of salts and organic solvents, require stabilization of multimeric enzymes [24,25]. Therefore, novel, multifunctional supports for enzyme immobilization, which are characterized by numerous different functional groups on their surfaces, are highly required. The presence of different moieties improves enzyme stability and reusability by formation of covalent bonds between the biomolecules and the support. Particularly, hydroxyl, epoxy, carbonyl and divinylsulfone groups are of high importance [26]. However, it should be mentioned that heterofunctional supports also possess disadvantages, as a specific pH value (approx. 10) is required in case of carbonyl groups to form covalent bonds [16]. Thus, hybrid oxide systems should be distinguished among the support materials due to the presence of numerous –OH groups onto their surface. Research carried out by Pezella et al., which included the use of perlite (mixture of SiO_2_, Al_2_O_3_, Na_2_O, K_2_O, Fe_2_O_3_, MgO and CaO) for immobilization of laccase and verification of this biocatalytic system in the process of decolorization of Remazol Brilliant Blue R dye, confirm the previously mentioned statement [27]. Nevertheless, only a few literature reports concerning the use of the aforementioned materials for immobilization of laccase and removal of dyes can be found to date. Hybrid oxide materials, such as SiO_2_–Fe_3_O_4_ [28,29], TiO_2_–ZrO_2_–SiO_2_ [30] or graphene oxide enriched by inorganic additives [31,32], were used for the removal of dyes from wastewater. It should also be mentioned that nanoparticles of ZnO/MnO_2_ chelated with Cu^2+^ were used as a support for laccase, and allowed to achieve more than 85% degradation of the Alizarin Red S dye [33]. Despite numerous studies regarding the immobilization of laccase on various supports, new support materials are still being sought for improvement of the effectiveness of biocatalytic processes. This fact is caused by the necessity to deeply understand the kinetics of pollutants degradation, in case of which the substrates consist of various phenolic compounds at different concentrations and various origins [34].

Taking the aforementioned information into account, the decolorization efficiency of Remazol Brilliant Blue R by laccase immobilized onto ZrO_2_–SiO_2_ and ZrO_2_–SiO_2_/Cu^2+^ materials was determined in the presented study. The use of zirconia–silica hybrid supports is determined by the need to search for more effective support materials and methods of enzyme immobilization, and hence, more efficient biocatalytic systems. Moreover, laccase immobilization onto the obtained porous oxide materials by adsorption did not alter the three-dimensional structure of the enzyme and may provide a stabilizing microenvironment for the biomolecules, and thus increase their catalytic activity and stability. Nevertheless, it should be mentioned that enzyme stabilization due to adsorption is challenging. This is associated with the blocking of pores of the support as well as the formation of physical interactions which lead to the partial inactivation of the enzyme, as suggested by dos Santos et al. [35]. Furthermore, the presence of –OH moieties and hydrophilic character of the produced supports may additionally positively affect enzyme–support interactions. In addition, the use of a laccase inducer, such as copper ions [36], allowed to compare the efficiency of the processes catalysed by this oxidoreductase with and without the presence of the metal ions. During the investigation, the solution of RBBR dye at a concentration of 50 mg/L was used in order to reflect the actual concentration of this dye in wastewater [37]. The effect of process duration, temperature and pH was investigated in order to determine the most suitable decolorization conditions. The obtained materials were extensively characterized using scanning electron microscopy, Fourier transform infrared spectroscopy, low-temperature N_2_ sorption and electrophoretic mobility measurements. Kinetic parameters, such as Michaelis–Menten constant (*K_m_*) and the maximum reaction rate (*V_max_*), were calculated. Furthermore, the amount of immobilized enzyme as well as reusability and storage stability of the novel biocatalytic systems were determined. The median lethal concentration (LC_50_) of *Artemia salina* microorganisms and their mortality were evaluated to compare the toxicity of dye solution before and after the decolorization processes.

## 2. Materials and Methods 

### 2.1. Chemicals and Materials

**Figure 1:**
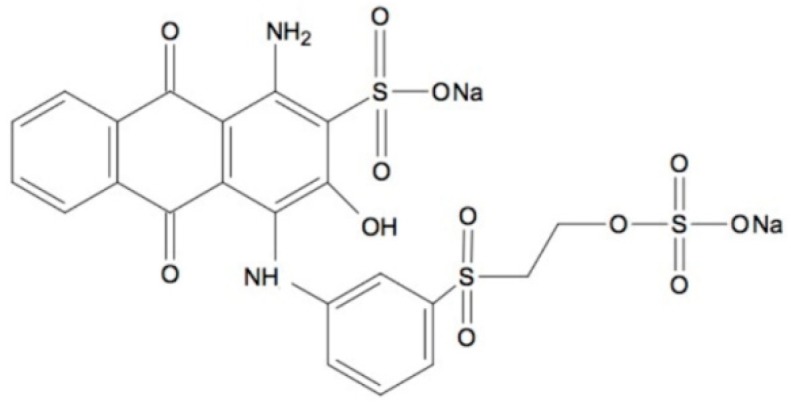
Chemical structure of the Remazol Brilliant Blue R dye (RBBR).

Zirconium(IV) isopropoxide, tetraetoxysilane, copper(II) sulfate pentahydrate, laccase from *Trametes versicolor* (EC 1.10.3.2.), Remazol Brilliant Blue R (RBBR), sodium acetate, ammonium and phosphate buffer solutions, potassium chloride, sodium chloride, Coomassie Brilliant Blue G-250 (CBB G-250), and 2,2-azinobis-3-ethylbenzothiazoline-6-sulfonate (ABTS) were obtained from Sigma-Aldrich (St. Louis, MO, USA). Isopropyl and ethyl alcohol, ammonia solution, hydrochloric acid (35–38%) and glacial acetic acid were purchased from the Chempur Company (Piekary Śląskie Poland). The chemical structure of the Remazol Brilliant Blue R dye is presented in Figure 1. 

### 2.2. Synthesis of ZrO_2_–SiO_2_ and ZrO_2_–SiO_2_/Cu^2+^ Oxide Systems

The ZrO_2_–SiO_2_ hybrid, with a molar ratio of precursors equal to 1.5:1, was synthesized via a modified sol–gel route, according to the previously published research [38]. The reactor (1 L) was filled with 500 mL of isopropyl alcohol. Simultaneous dosing of 120 mL of zirconium(IV) isopropoxide and 90 mL of tetraetoxysilane using ISM833A peristaltic pumps (ISMATEC, Wertheim, Germany) was the crucial step of the synthesis. After 1 h of stirring (Eurostar Digital stirrer, Ika Werke GmbH, Staufen im Breisgau, Germany), 60 mL of 25% solution of ammonia (promoter of hydrolysis), was added dropwise. The mixture was additionally stirred within 1 h. After the specified reaction time, the reactor content was placed in a fume hood until a gel was obtained (24 h). Then, the obtained material was slowly dried at 105 °C within 24 h. In order to obtain ZrO_2_–SiO_2_/Cu^2+^, the classified material was modified with copper (II) ions. Briefly, 2.5 g of ZrO_2_–SiO_2_ hybrid was placed into a round bottom flask together with 10 mL of 10% copper (II) sulfate solution. After 1 h of stirring, the mixture was placed in a vacuum evaporator in order to remove the solvent. The obtained materials were comprehensively analyzed and used as supports in the immobilization process of laccase.

### 2.3. Immobilization of Laccase 

In order to immobilize laccase onto the obtained materials, 100 mg of the ZrO_2_–SiO_2_ or ZrO_2_–SiO_2_/Cu^2+^ hybrids were placed into vials (20 mL). In the next step, 10 mL of laccase solution at pH 5 (sodium acetate buffer) and concentration of 1 mg/mL was added. The immobilization was carried out using an IKA KS 4000i control incubator (Ika Werke GmbH, Staufen im Breisgau, Germany) at 25 °C for 1 h. After incubation, the obtained biocatalytic systems were centrifuged using a LLG uniCFUGE 5 (LLG Labware, Dublin, Ireland) at 4000 rpm and washed several times with sodium acetate buffer (pH 5).

### 2.4. Storage Stability and Kinetic Measurements of Free and Immobilized Laccase

In order to define the storage stability of free and immobilized enzyme and to calculate the Michaelis–Menten constant (*K_m_*) and maximum rate of reaction (*V_max_*), investigations were conducted according to the previously published study [30]. The measurements were carried out using ABTS (maximum absorbance at *λ* = 420 nm) as the model substrate. Briefly, free and immobilized biocatalysts were stored in sodium acetate buffer solution (pH 5) at 4 °C within 20 days in order to examine their storage stability. The V-750 spectrophotometer (Jasco, Tokio, Japan) was used to investigate changes in substrate concentration after the catalytic reaction. Based on the results, storage stability was estimated and relative activity was calculated using the following Equation (1):

(1) $$Relative\;activity\;\left(\%\right)=\frac{A_{I}}{A_{0}}\cdot 100\%$$

where *A*_0_ denotes the initial activity of the laccase, and *A_I_* denotes the activity of the immobilized enzyme.

The oxidation reaction of ABTS was used to calculate the Michaelis–Menten constant (*K_m_*) and the maximum rate of reaction (*V_max_*), based on Hanes–Wolf plot. Kinetic parameters were evaluated under optimal reaction conditions using substrate solutions at various concentrations.

### 2.5. Decolorization of Remazol Brilliant Blue R Dye

In order to determine the sorption properties of oxide materials prior to dye decolorization, the experiments were carried out using both biocatalytic systems with a thermally inactivated enzyme. The obtained heterogeneous biocatalysts were placed in an IKA KS 4000i control incubator (Ika Werke GmbH, Staufen im Breisgau, Germany) at 80 °C for 2 h, in order to deactivate the enzyme. Furthermore, 100 mg of each of the materials with the inactivated enzyme were placed in the vials together with 10 mL of Remazol Brilliant Blue R dye at a concentration of 50 mg/L (pH 4, 30 °C, 24 h). After a specified period of time, the absorbance of the resulting solution was measured.

To establish the effect of process duration on decolorization efficiency, the experiments were performed at 30 °C and pH 4 using dye solution at the concentration of 50 mg/L. The samples were collected after 0.5, 1, 3, 5, 8, 12 and 24 h of the decolorization process. The influence of temperature, ranging from 10 to 70 ℃, on decolorization efficiency was examined using 10 mL of dye solution at the concentration of 50 mg/L (pH 4, 24 h). The effect of pH on the dye removal was also examined using 10 mL of dye solution at the concentration of 50 mg/L, at 30 °C and at wide pH range from 2 to 10.

Due to the fact that immobilized enzymes should be characterized by good reusability, the biocatalytic systems produced were tested over seven consecutive catalytic cycles. Briefly, after 24 h of decolorization reaction, the obtained heterogeneous biocatalysts were centrifuged using a 5810 R centrifuge (Eppendorf, Hamburg, Germany) at 4000 rpm and washed with sodium acetate buffer at pH 4 to remove dye molecules. The materials prepared in this way were used once again. The reusability study was performed under optimal process conditions which allowed for the highest removal efficiency of the dye (pH 4, 30 °C and 24 h). Each experiment was carried out in triplicate and the results are presented as an average value.

### 2.6. Analytical Techniques

In order to describe the morphology of the ZrO_2_–SiO_2_ and ZrO_2_–SiO_2_/Cu^2+^ oxide materials before and after immobilization of laccase, SEM images were obtained using EVO40 apparatus (Zeiss, Berlin, Germany). FTIR spectra were obtained using a Vertex 70 spectrometer (Bruker, Billerica, MA, USA). Samples were prepared in the form of pellets by mixing 1.5 mg of the analyzed material with 200 mg of anhydrous KBr. ASAP 2020 physisorption analyzer (Micromeritics Instrument Co., Norcross, GA, USA) was used in order to determine the parameters of the porous structure of synthesized materials, such as Brunauer–Emmett–Teller (BET) surface area, meanwhile mean pore size and total pore volume were calculated based on the Barrett–Joyner–Halenda (BJH) method. Before measurement, ZrO_2_–SiO_2_ and ZrO_2_–SiO_2_/Cu^2+^ hybrids and biocatalytic systems were degassed at 120 °C within 4 h. Furthermore, they were subjected to analysis using low-temperature (−196 °C) sorption of N_2_. The electrokinetic stability of the materials was investigated using an Zetasizer Nano ZS instrument equipped with an MPT-2 autotitrator (Malvern Instruments Ltd., Malvern, United Kingdom). The samples were prepared by dispersing 10 mg of the oxide material in 25 mL of a 0.001 M NaCl solution. The amount of laccase immobilized onto ZrO_2_–SiO_2_ and ZrO_2_–SiO_2_/Cu^2+^ oxide materials was calculated based on the Bradford method [39]. The UV-Vis spectroscopy was used to measure the changes in the absorbance of the RBBR during the decolorization process and to calculate the degradation efficiency. The measurements were carried out at wavelength equal to 592 nm (*λ_max_* of RBBR dye) using a V-750 spectrophotometer (Jasco, Tokio, Japan). The decolorization efficiencies were calculated using Equation (2):

(2) $$DDE\;\left(\%\right)=\frac{C_{B}-\;C_{A}}{C_{B}}\cdot 100\%$$

where *DDE* (%) denotes RBBR dye decolorization efficiency, *C_B_* and *C_A_* denote RBBR dye concentration before and after decolorization process, respectively.

**Figure 2:**
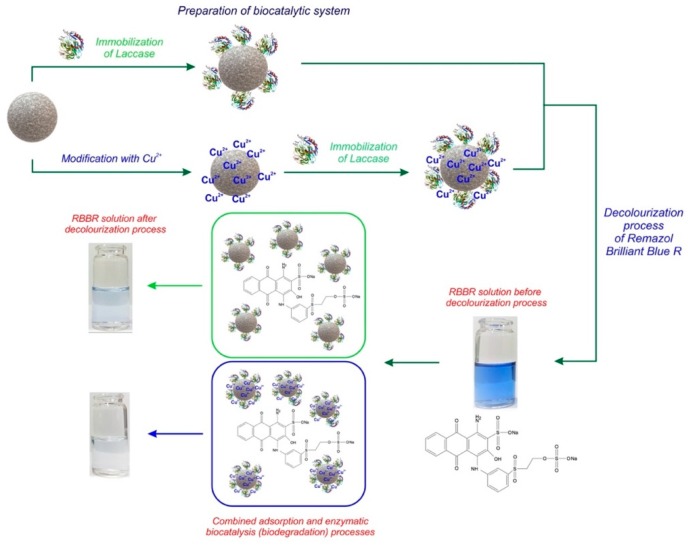
Schematic diagram of preparation of biocatalytic systems and decolorization process of Remazol Brilliant Blue R (RBBR) dye.

The idea of the investigations is presented in Figure 2.

Toxicity study of the RBBR dye solution before and after decolorization was carried out using the *Artemia salina* test microorganism, according to the previously published study [40]. Briefly, 0.5 g of *Artemia salina* eggs were incubated in 500 mL of NaCl solution at a concentration of 25 g/L (25 °C with exposure to permanent lighting for 24 h). After that time, 10 larvae were placed into a specified sample and left for 24 h at 25 °C. The percentage of mortality of *Artemia salina* was calculated for the reaction media before and after the enzymatic treatment of dyes. The calculation of number of dead larvae was conducted and median lethal concentration (LC_50_) was defined. The tests were carried out in triplicate.

## 3. Results and Discussion

### 3.1. Characterization of the Oxide Materials before and after Immobilization of Laccase 

**Figure 3:**
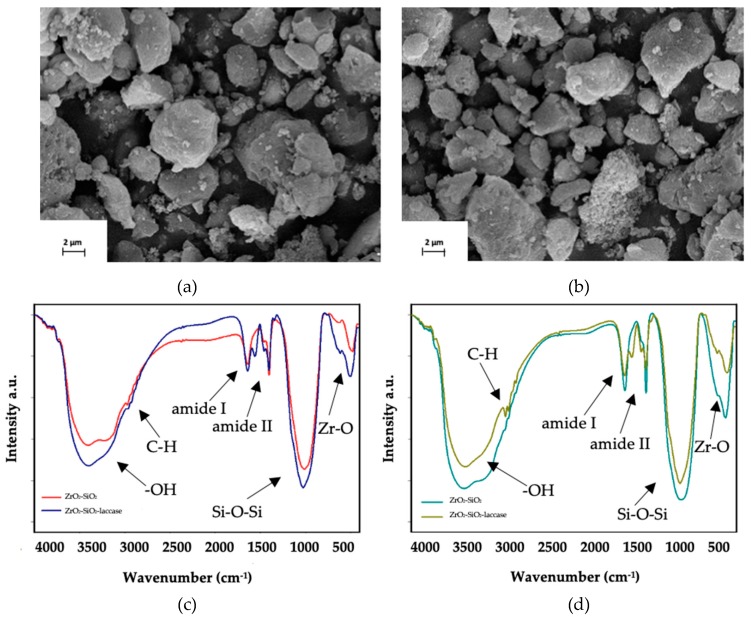
Scanning electron microscope (SEM) images of: (**a**) ZrO_2_–SiO_2_ and (**b**) ZrO_2_–SiO_2_/Cu^2+^ and FTIR spectra of: (**c**) ZrO_2_–SiO_2_, ZrO_2_–SiO_2_–laccase and (**d**) ZrO_2_–SiO_2_/Cu^2+^ and ZrO_2_–SiO_2_/Cu^2+^–laccase systems.

SEM and FTIR spectral analyses were performed in order to confirm both the effective synthesis of ZrO_2_–SiO_2_ and ZrO_2_–SiO_2_/Cu^2+^ oxide systems and immobilization of laccase onto the obtained materials (Figure 3). The morphology of materials was evaluated based on SEM images, presented in Figure 3a,b, respectively. The obtained materials are characterized by irregular particles, 5 μm in diameter, which tend to aggregate. It is worth noticing that there were no significant changes in the structure of oxide material before and after doping with Cu^2+^.

The FTIR spectra of ZrO_2_–SiO_2_ material and ZrO_2_–SiO_2_/Cu^2+^ before and after immobilization of laccase are presented in Figure 3c,d. The spectrum of ZrO_2_–SiO_2_ possessed a wide band assigned to stretching vibrations of –OH groups between 3650 and 3350 cm^−1^ and a signal at 1630 cm^−1^ characteristic for bending vibrations of physically adsorbed water. The signal at 1480 cm^−1^, which is characteristic for deformational vibrations of –NH group, presumably resulted from the use of ammonia during synthesis of the oxide material. The most characteristic groups in the structure of the obtained material are represented by the peaks with maxima at 1330 cm^−1^ (Zr-OH bond), 1200–950 cm^−1^ (Si-O-Si and Zr-O-Zr bonds), 675 cm^−1^ (Si-O bond) and 600 cm^−1^ (Zr-O bond) [38,41]. The FTIR spectrum of the ZrO_2_–SiO_2_–laccase system included signals, which can be seen at 1625, 1555 and 1250 cm^−1^, assigned to the hybrid oxide system as well as laccase. These signals were attributed to the stretching vibrations of amide I, II and III bonds, respectively, and their presence indicates an effective deposition of enzyme molecules onto oxide material surface [21]. The FTIR spectra of ZrO_2_–SiO_2_/Cu^2+^ and ZrO_2_–SiO_2_/Cu^2+^–laccase materials (Figure 3d), included the same signals as those presented in the spectra of ZrO_2_–SiO_2_ and ZrO_2_–SiO_2_–laccase systems. However, particular attention should be paid to the signal at wavenumber of 601 cm^−1^, which corresponds to vibrations of Cu-O bonds and confirms efficient doping of oxide material with Cu^2+^ ions [42]. Thus, based on the FTIR results, the effective synthesis and functionalization of the oxide materials as well as successful enzyme immobilization have been confirmed.

**Figure 4:**
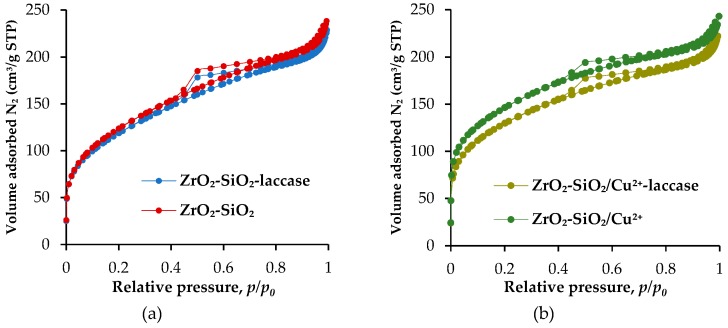
Low temperature N_2_ adsorption/desorption isotherms of: (**a**) ZrO_2_–SiO_2_, ZrO_2_–SiO_2_–laccase and (**b**) ZrO_2_–SiO_2_/Cu^2+^ and ZrO_2_–SiO_2_/Cu^2+^–laccase systems.

The results of low-temperature N_2_ adsorption/desorption for materials before and after immobilization process are presented in Figure 4. Each of the illustrated isotherms (for ZrO_2_–SiO_2_, ZrO_2_–SiO_2_–laccase, ZrO_2_–SiO_2_/Cu^2+^ and ZrO_2_–SiO_2_/Cu^2+^–laccase systems) was classified as type IV with type H4 hysteresis loops. The calculated values of BET surface area, the total pore volume and the mean pore diameter are presented in Table 1. The parameters of the porous structure of the materials before laccase immobilization showed that the analyzed samples possessed higher surface area and total pore volume compared to the ZrO_2_–SiO_2_–laccase and ZrO_2_–SiO_2_/Cu^2+^–laccase systems. These results confirm the effective enzyme immobilization onto the surface and inside the pores of the synthesized oxide materials.

**Figure 5:**
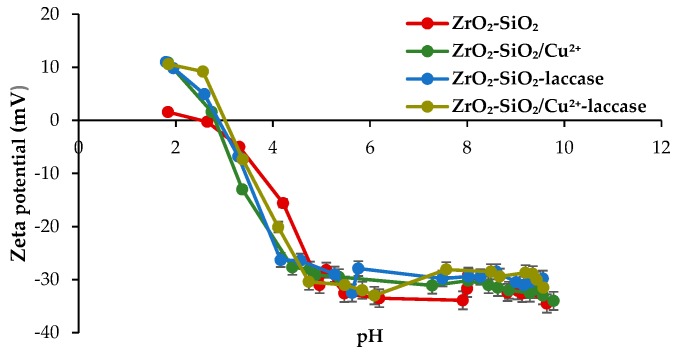
Electrokinetic curves of the synthesized materials and biocatalytic systems.

The zeta potential is an important feature for the evaluation of the surface properties of the oxide materials [43]. The obtained results (zeta potential vs. pH) for both pure and Cu^2+^-doped materials before and after the immobilization process are presented in Figure 5. It can be observed that the zeta potential values of all samples are negative in the analyzed pH range. In addition, it can be concluded that the zeta potential strongly depends on the pH value. Beyond the isoelectric point (IEP), which is almost the same for each of the samples and equal to 3, the zeta potential values become positive. Moreover, it should be underlined that the zeta potential after functionalization and enzyme immobilization is almost unaltered, due to the relatively low amount of Cu^2+^ ions or laccase which were deposited onto the surface of oxide system. Nevertheless, values of IEP close to 3 were mainly caused by the presence of silica in the oxide material, the IEP of which is located between 2 and 3 [44,45]. However, the slight shift of isoelectric point values towards higher pH for samples after laccase immobilization indicated that the attached enzyme also affected the electrokinetic properties of the analyzed samples as laccase possesses two IEP, the first one at approx. pH 3 and the second in a pH range of 4.6–6.8, as previously reported by Jolivalt et al. [46]. Moreover, each of tested systems possessed higher stability in neutral and alkaline environment (pH above 6).

The kinetic parameters of free and immobilized enzymes as well as efficiency of laccase immobilization onto ZrO_2_–SiO_2_ and ZrO_2_–SiO_2_/Cu^2+^ were also calculated. The immobilization yield obtained for hybrid and Cu^2+^-doped oxide systems reached 86% and 94%, respectively, which corresponds to effective enzyme immobilization onto both supports (Table 2). The high immobilization yields were mainly associated with the high porosity of the supports and the presence of numerous hydroxyl groups on their surface, which facilitate the formation of hydrogen bonds and electrostatic interactions between the biocatalyst and surface of the oxide systems [47]. 

Kinetic parameters, such as the Michaelis–Menten constant (*K_m_*) and the maximum reaction rate (*V_max_*), were also calculated and presented in Table 2. The higher values of *K_m_* and lower values of *V_max_* noticed for immobilized laccase (in comparison to free laccase) indicated its lower substrate affinity and lower reaction rate, respectively. Lower substrate affinity of the immobilized enzymes might be related to the conformational changes in the enzyme structure upon immobilization, as lower maximum reaction rate could be explained by the formation of substrate diffusional limitations after enzyme binding. However, blocking of the enzymes active sites by the substrate or products of the reaction cannot be excluded [48]. It is worth noticing that the oxide matrix doped with copper ions showed a slight decrease of *K_m_* value and a slight increase of *V_max_* value, in comparison to the system without Cu^2+^, which implies the positive effect of these ions on the kinetic parameters of immobilized laccase. The obtained data are in agreement with the results of catalytic activity retention, as laccase immobilized onto ZrO_2_–SiO_2_ and ZrO_2_–SiO_2_/Cu^2+^ retained 65 and 76% of free enzyme activity, respectively. 

**Figure 6:**
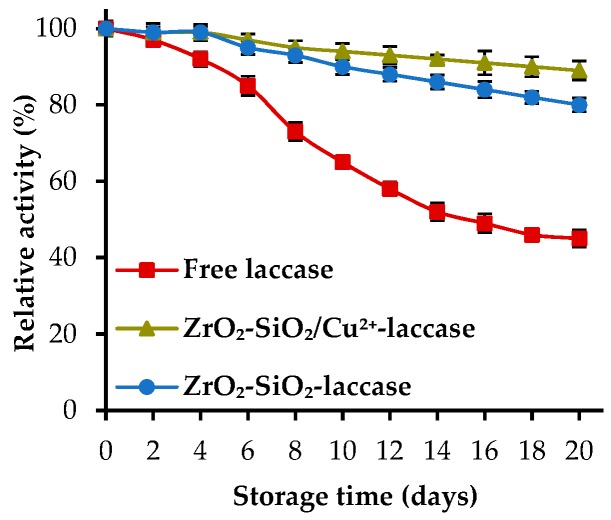
Storage stability of the free and immobilized enzyme over 20 days of storage.

Storage stability is one of the crucial parameters which indicate the possibility to use the immobilized enzymes at an industrial scale. The investigation of the storage stability of the free and immobilized laccases was carried out over 20 days (Figure 6). After 20 days of storage, laccase deposited onto ZrO_2_–SiO_2_ and ZrO_2_–SiO_2_/Cu^2+^ exhibited definitely higher catalytic activity (over 80%), as compared to the native enzyme (43%). However, a slight difference between the relative activity of laccase immobilized onto initial and Cu^2+^-doped oxide system can be observed. The enzyme deposited onto material doped with copper ions retained approx. 90% activity, as laccase immobilized onto ZrO_2_–SiO_2_ possessed only 82% of its initial activity after 20 days of storage. The higher activity of the enzyme immobilized onto both oxide systems might be explained by the stabilization of the biocatalyst structure upon immobilization and protective effect of the support material, which was also shown in previously published studies [30]. Nevertheless, the higher relative activity of the laccase immobilized onto Cu^2+^-doped oxide material is directly associated with the additional reinforcement of the enzyme structure by the metal ions [48]. The presented results correspond well with the data published by Fu et al., regarding the degradation of bisphenol A using laccase–copper phosphate hybrid nanoflowers. After 60 days of storage, the immobilized biocatalysts retained 90% of its initial activity [49]. In another study, presented by Batule et al., the laccase nanoflowers synthesized with copper phosphate significantly enhanced enzyme stability, which exceeded 95% after 1 month of storage. Moreover, after that time free laccase demonstrated only approx. 20% of its initial activity [50]. These results confirm the stabilizing effect of copper ions towards laccase structure and its activity.

### 3.2. Decolorization of Dye

#### 3.2.1. Decolorization of Dye Using Oxide Materials with Inactivated Enzyme

The first step of investigations concerning the dye decolorization process involved testing of the sorption capacities of ZrO_2_–SiO_2_ and ZrO_2_–SiO_2_/Cu^2+^ oxide systems toward RBBR. For this purpose, supports with thermally inactivated enzyme were placed into solution of the dye at concentration of 50 mg/L. The results showed that sorption efficiencies were equal to 6.38% and 0.52% for the ZrO_2_–SiO_2_–laccase and ZrO_2_–SiO_2_/Cu^2+^–laccase, respectively. Since the error values of the measurements were estimated at 3%, it can be concluded that sorption of the dye onto the obtained materials was negligible. These results can be compared with our previous study [30], in which the sorption efficiencies of the RBBR dye from solution at concentration of 10 mg/L for oxide materials such as TiO_2_–ZrO_2_ and TiO_2_–ZrO_2_–SiO_2_ were equal to 46% and 28%, respectively.

#### 3.2.2. Effect of Process Duration on Decolorization Efficiency

**Figure 7:**
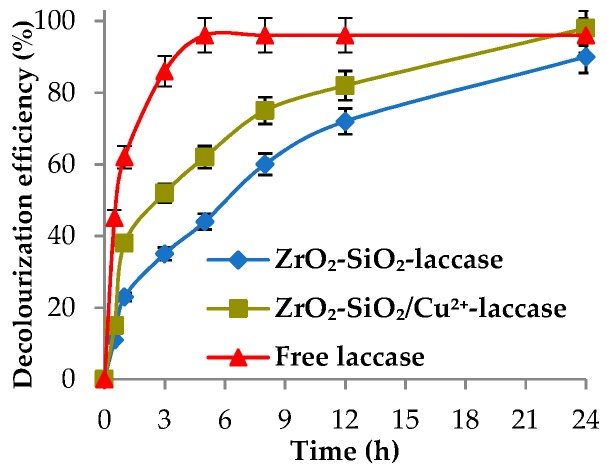
Effect of process duration on decolorization efficiency of the RBBR dye using free laccase and synthesized biocatalytic systems.

After determination of the sorption capacities of oxide materials with inactivated enzymes, the effect of the process duration on decolorization efficiency of RBBR dye by the immobilized enzyme was investigated. The obtained results are presented in Figure 7.

**Figure 8:**
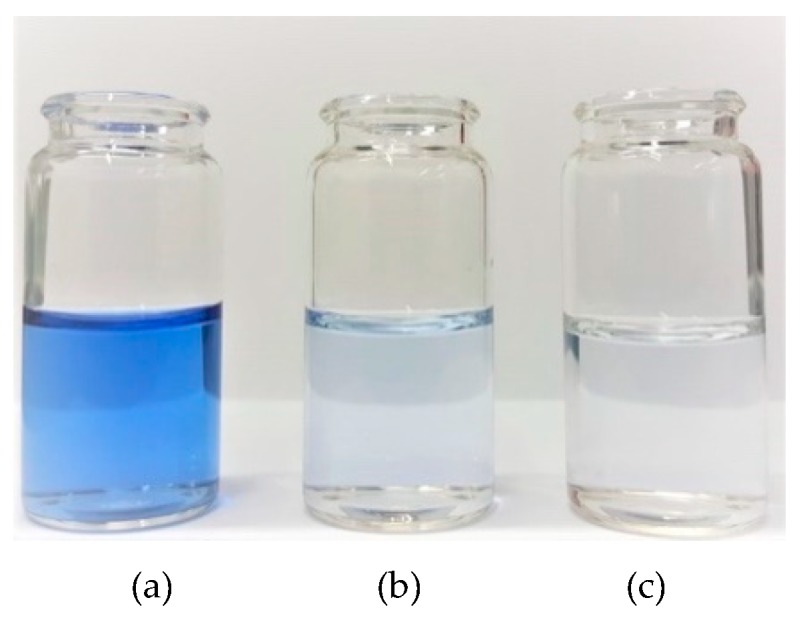
The solutions of the RBBR dye: (**a**) at initial concentration of 50 mg/L, (**b**) after decolorization using ZrO_2_–SiO_2_–laccase and (**c**) after decolorization using ZrO_2_–SiO_2_/Cu^2+^–laccase. Each of experiment was conducted at 30 °C and pH 4 within 24 h.

It can be observed that application of the biocatalytic system doped with copper ions resulted in higher decolorization efficiency of the RBBR dye over the analyzed process duration, as compared with biocatalytic systems without metal ions. Clearly, after 1 h of process, the decolorization efficiency reached 39% and 22% using the ZrO_2_–SiO_2_–laccase system with and without Cu^2+^, respectively. After 24 h, the efficiency of the decolorization process of the RBBR dye by biocatalytic system with copper ions was equal to 98%, as the removal rate of the dye by the ZrO_2_–SiO_2_–laccase system reached 90%. The solutions of the RBBR dye before and after enzymatic decolorization processes were presented in Figure 8. Based on the results, it was confirmed that 24 h is the most suitable time, because its prolongation does not increase the biodegradation efficiencies, irrespectively of the biocatalytic system used. It should be noted that the decolorization efficiency reached 96% using a native enzyme after 24 h of the process at pH 5 and temperature of 30 °C. The difference between decolorization efficiencies using ZrO_2_–SiO_2_ and ZrO_2_–SiO_2_/Cu^2+^ systems resulted from the presence of Cu^2+^ ions, which positively affected the activity and stability of the laccase [51,52]. In another publication, Bayramoglu et al. showed that the presence of transition metal ions, such as Cu^2+^, on the surface of the support obtained from poly(4-vinylpyridine) molecules on the magnetic beads is responsible for strong binding between pyridine ring/Cu^2+^ and laccase [53]. This may explain the higher efficiency of decolorization by laccase immobilized onto ZrO_2_–SiO_2_/Cu^2+^ support. Similar observations were made by Olajuyigbe et al. which indicated that laccase immobilized onto Cu–alginate beads shows higher relative activity, as compared to native enzyme and calcium–alginate support [54,55]. 

#### 3.2.3. Effect of pH and Temperature on Decolorization Efficiency

**Figure 9:**
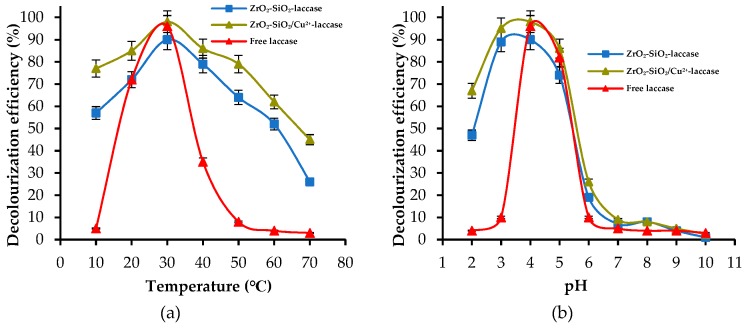
Effect of: (**a**) temperature and (**b**) pH of dye solution on decolorization efficiencies.

The next step of investigations was focused on the evaluation of the influence of the pH and temperature of dye solution on the decolorization efficiency. These parameters play a crucial role due to the relatively low stability of enzymes under harsh reaction conditions [34]. The effect of temperature was examined in the range from 10 to 70 °C at pH 4, whereas the effect of pH was evaluated in the range from 2 to 10 at 30 °C using the RBBR dye solution at the concentration of 50 mg/L (Figure 9).

In the analyzed temperature range, the decolorization process occurred with higher efficiencies when the dye was degraded in a system containing copper ions. It is worth mentioning that the most significant difference between the removal rates of the dye obtained using both types of materials was equal to approx. 25% at 10 °C. Nevertheless, the highest values of dye removal were obtained at 30 °C, irrespectively of the biocatalytic system used. It should also be added that even at 60 °C both systems after immobilization allowed for the removal of more than 50% of the dye. However, increase of the temperature to 70 °C caused a decrease of the decolorization efficiency, which reached 26% for ZrO_2_–SiO_2_–laccase and 45% for ZrO_2_–SiO_2_/Cu^2+^–laccase, respectively. This fact may be related to the thermal inactivation of the enzyme. The obtained results were compared with the data presented by Tapia-Orozco et al., which were focused on laccase immobilized onto TiO_2_ particles. The obtained biocatalytic system was tested at 80 °C and showed a lack of enzymatic activity [56]. Conclusion can be drawn based on the analysis of the results which show effect of pH on the decolorization efficiency (Figure 9b). Over the tested pH range, the removal rates were higher when the ZrO_2_–SiO_2_/Cu^2+^–laccase system was used. Moreover, the highest decolorization efficiency was achieved at pH 4 for both systems. Significant decrease of the decolorization efficiency was noticed when the process was realized at the pH range of 6–10. This may result from the reduction of catalytic activity of laccase in the near-neutral and basic environment due to conformational rearrangements in the structure of biomolecule, caused by changes in the nature of aminoacid groups [57]. 

#### 3.2.4. Reusability of the Biocatalytic Systems

**Figure 10:**
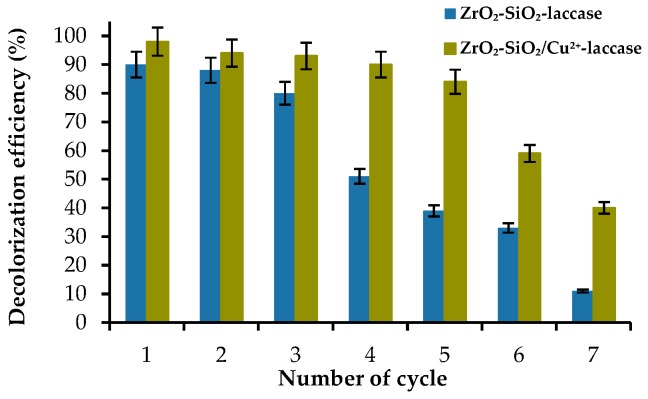
Reusability of the obtained biocatalytic systems.

Reusability as well as storage stability are important parameters which need to be considered when immobilized enzymes are used at an industrial scale. The possibility to use the biocatalytic systems multiple times possesses many advantages, the most important among which is the reduction of process costs [58]. In the presented study, the decolorization efficiency of the RBBR dye by ZrO_2_–SiO_2_–laccase and ZrO_2_–SiO_2_/Cu^2+^–laccase systems was tested over seven successive decolorization cycles (Figure 10).

The decolorization efficiency constantly decreased over the catalytic cycles. However, the more prominent decrease of the removal rates can be observed from the 4^th^ catalytic cycle, particularly in case of ZrO_2_–SiO_2_–laccase system (decrease from 77% in the 3^rd^ cycle to 50% in the 4^th^ cycle). It may be explained by the elution of laccase from the support, which has been also shown in our previous study [30]. After seven consecutive catalytic cycles, the results showed that approx. 40% and 10% of the RBBR dye was removed by the ZrO_2_–SiO_2_/Cu^2+^–laccase system and system without copper ions, respectively. The decrease in enzyme activity might be related to the inhibition of the enzyme by the reaction products and partial deactivation of the biocatalyst. It is worth noticing that significant differences between the degradation efficiency of the RBBR dye after treatment with ZrO_2_–SiO_2_–laccase and ZrO_2_–SiO_2_/Cu^2+^–laccase over seven catalytic cycles might be related to the stabilizing effect of copper ions on laccase activity as well as the fact that Cu^2+^ ions act as an inducer for the transfer of electrons which helps overcome enzyme inactivation [36]. In another study, Chen et al. immobilized laccase onto magnetic graphene oxide and used this system for degradation of dyes such as Crystal Violet, Malachite Green and Brilliant Green. After 10 catalytic cycles, the relative activity of the obtained biocatalytic system reached approx. 60% [59]. On the other hand, Le et al. showed that laccase encapsulated in core–shell magnetic copper alginate beads removed only approx. 20% of the RBBR dye after three consecutive decolorization cycles [60]. These results clearly show that type of the support material strongly affects both laccase activity and decolorization efficiency.

### 3.3. Toxicity Study

**Figure 11:**
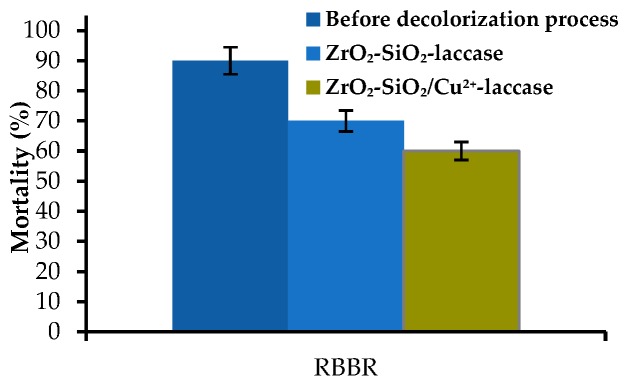
Mortality of *Artemia salina* in the solution before and after enzymatic treatment.

In order to determine the lethal concentration of the RBBR dye, solutions of the dye at different concentrations, ranging from 0.5 to 50 mg/L, were tested. It was evaluated that the LC_50_ of the RBBR dye before enzymatic treatment towards *Artemia salina* larvae as a model microorganism is equal to 16 mg/L. Moreover, the mortality of the tested microorganisms was lower after the decolorization process (pH 4, 30 °C and 24 h) by 20 and 30% for ZrO_2_–SiO_2_–laccase and ZrO_2_–SiO_2_/Cu^2+^–laccase, respectively, as compared to dye solution before enzymatic treatment (Figure 11). 

In another study by da Silva et al., horseradish peroxidase was used for removal of RBBR from an aqueous solution. Authors noticed that enzymatic treatment caused a reduction of mortality of *Artemia salina* up to 45% as compared to the solution before the decolorization [61]. The higher mortality data presented in this work might indicate the formation of RBBR degradation products which exhibit higher toxicity as compared to the RBBR degradation products generated using laccase [62]. That fact is closely related to the different mechanism of catalytic behaviour of laccase and horseradish peroxidase.

## 4. Conclusions

In this study the hybrid ZrO_2_–SiO_2_ and ZrO_2_–SiO_2_/Cu^2+^ oxide systems were used as supports for laccase immobilization and further as an effective biocatalytic system for the decolorization processes of the Remazol Brilliant Blue R dye. Results of scanning electron microscopy and Fourier transform infrared spectroscopy confirmed the effective synthesis of the oxide systems and efficient laccase immobilization. Analysis of the effect of various decolorization parameters on its efficiency showed that the highest removal rates of RBBR, equal 98% and 90% for ZrO_2_–SiO_2_–laccase and ZrO_2_–SiO_2_/Cu^2+^–laccase, respectively, were observed after 24 h of the process at 30 ℃ and pH 4. Thus, the obtained results show that addition of copper ions into the support material has a positive impact on laccase stability and activity. Additionally, the toxicity of the dye solution after enzymatic treatment using ZrO_2_–SiO_2_–laccase and ZrO_2_–SiO_2_/Cu^2+^–laccase was reduced by approx. 20% and 30%, respectively, as compared to initial RBBR dye solution. However, due to slight decrease of toxicity of the solution after laccase treatment, the use of another enzyme, such as horseradish peroxidase, could be considered. Nevertheless, analysis of the results allows to conclude that effective enzymatic systems were obtained, which may potentially be applied for the removal of dyes. Moreover, it should be noted that the obtained oxide materials might be used as supports for the immobilization of a wide range of catalytic proteins. These biocatalytic systems may be also used for the degradation processes of other contaminants, e.g., bisphenols or pharmaceuticals. 

## Figures and Tables

**Table 1:** Parameters of the porous structure of the obtained materials before and after immobilization of laccase.

Examined System	Parameter		
	*A_BET_* (m^2^/g)	*V_p_* (cm^3^/g)	*S_p_* (nm)
ZrO_2_–SiO_2_	440.2	0.369	3.4
ZrO_2_–SiO_2_–laccase	419.9	0.354	3.4
ZrO_2_–SiO_2_/Cu^2+^	498.3	0.376	3.0
ZrO_2_–SiO_2_/Cu^2+^–laccase	445.7	0.344	3.0

**Table 2:** Kinetic parameters of free and immobilized enzyme, amount of immobilized enzyme and immobilization yield.

Kinetic Parameters and Immobilization Data	Free Laccase	ZrO_2_–SiO_2_–Laccase	ZrO_2_–SiO_2_/Cu^2+^–Laccase
*K_m_* (mM)	0.049 ± 0.002	0.132 ± 0.009	0.098 ± 0.008
*V_max_* (U/mg)	0.041 ± 0.006	0.029 ± 0.007	0.037 ± 0.009
Amount of enzyme (mg/g)	-	86 ± 3.8	94 ± 3.2
Immobilization yield (%)	-	86 ± 3.9	94 ± 3.1
